# Wingless and Archipelago, a fly E3 ubiquitin ligase and a homolog of human tumor suppressor FBW7, show an antagonistic relationship in wing development

**DOI:** 10.1186/s12861-020-00217-1

**Published:** 2020-06-29

**Authors:** Sujin Nam, Kyung-Ok Cho

**Affiliations:** Department of Biological Sciences, Korea Advanced Institute of Science and Technology, 291 Daehak-ro, Yuseong-gu, Daejeon, Korea

**Keywords:** Archipelago, Ago, FBW7, Chemosensory bristles, Shaft, Wg signaling, Wg secretion, E3 ubiquitin ligase

## Abstract

**Background:**

Archipelago (Ago) is a *Drosophila* homolog of mammalian F-box and WD repeat domain-containing 7 (FBW7, also known as FBXW7). In previous studies, FBW7 has been addressed as a tumor suppressor mediating ubiquitin-dependent proteolysis of several oncogenic proteins. Ubiquitination is a type of protein modification that directs protein for degradation as well as sorting. The level of beta-catenin (β-cat), an intracellular signal transducer in Wnt signaling pathway, is reduced upon overexpression of FBW7 in human cancer cell lines. Loss of function mutations in FBW7 and overactive Wnt signaling have been reported to be responsible for human cancers.

**Results:**

We found that Ago is important for the formation of shafts in chemosensory bristles at wing margin. This loss of shaft phenotype by knockdown of *ago* was rescued by knockdown of *wingless* (*wg*) whereas wing notching phenotype by knockdown of *wg* was rescued by knockdown of *ago*, establishing an antagonistic relationship between *ago* and *wg.* In line with this finding, knockdown of *ago* increased the level of Armadillo (Arm), a homolog of β-cat, in *Drosophila* tissue. Furthermore, knockdown of *ago* increased the level of Distal-less (Dll) and extracellular Wg in wing discs. In S2 cells, the amount of secreted Wg was increased by knockdown of Ago but decreased by Ago overexpression. Therefore, Ago plays a previously unidentified role in the inhibition of Wg secretion. Ago-overexpressing clones in wing discs exhibited accumulation of Wg in endoplasmic reticulum (ER), suggesting that Ago prevents Wg protein from moving to Golgi from ER.

**Conclusions:**

We concluded that Ago plays dual roles in inhibiting Wg signaling. First, Ago decreases the level of Arm, by which Wg signaling is downregulated in Wg-responding cells. Second, Ago decreases the level of extracellular Wg by inhibiting movement of Wg from ER to Golgi in Wg-producing cells.

## Background

Degradation of proteins is a fast and reliable process to eliminate activity of a given protein spatiotemporally for various functions such as cell proliferation, differentiation and survival. Ubiquitination is one of major mechanisms for protein degradation by conjugating ubiquitin to a target protein that acts as a recruiting signal for proteasome complex [1]. In addition, ubiquitin moiety functions in proteasome-independent ways as an internalization signal or as a sorting signal to regulate endocytosis and secretory pathway [2]. Fly Ago is a component of F-box protein in E3 ubiquitin ligase complex, and it recognizes specific substrates such as Myc, Trachealess (Trh), Similar (Sima) and Cyclin E (Cyc E) for ubiquitination [3–6]. Similarly, human FBW7 as a homolog of Ago has over 60 known substrates such as Cyc E, Myc, Jun, Notch and mTOR [7–11]. FBW7 has been considered as a tumor suppressor and is one of the most frequently mutated genes in human cancers [12].

Wg/Wnt, as one of key signaling proteins, is highly conserved in all animals. It is involved in diverse cellular processes during development as well as adult homeostasis [13, 14]. It has been reported that fly Wg is secreted initially to the apical surface, transcytosed and then secreted to the basolateral surface in wing discs, which is consistent with findings that intracellular Wg is enriched in the apical region of Wg-producing cells but the majority of extracellular Wg is basolaterally enriched [15, 16]. In canonical Wg pathway, Wg binds to cell surface receptor Frizzled (Fz) and co-receptor Arrow (Arr), and their interaction leads to the stabilization of cytoplasmic Arm that moves into the nucleus and functions as a co-transcription factor [17], by which transcription of multiple genes such as *distal-less* (*dll*) is induced [18]. Overactivation of Wnt signaling is responsible for human cancers, especially colorectal cancer [19, 20].

Here, we show that Ago decreased the amount of Arm in wing discs, which is consistent with a report that FBW7 downregulates Wnt signaling by reducing the amount of β-cat [21]. Furthermore, knockdown of Ago increased the level of extracellular Wg in both wing discs and S2 cell culture. In contrast, overexpression of Ago decreased the level of secreted Wg in S2 cell culture. In line with this, Wg was accumulated in ER of wing disc cells upon Ago overexpression, suggesting problems in Wg trafficking. Thus, Ago downregulates Wg signaling by reducing the level of extracellular Wg in Wg-producing cells as well as by reducing the level of Arm in Wg-responding cells. Such cumulative effects of Ago on Wg signaling would affect Dll expression, wing size and development of chemosensory bristles.

## Results

### Ago is involved in wing growth and formation of chemosensory bristles

*ago* was identified as a modifier of *wg* in a genetic screen (Nam S., in preparation), which led us to examine the loss of *ago* phenotypes in the adult wing, a great tool for studying Wg signaling [22]. To modulate the level of Ago in flies, we utilized two *UAS-ago* lines (*myc-ago* and *ago*^*UAS.ORF*^) and two *UAS-ago RNAi* lines (*ago*^*HM04005*^ and *ago*^*HMS00111*^). These two *UAS-ago RNAi* lines target different regions in the *ago* gene (Additional file: Fig. S1A). These flies were all obtained from stock centers except for *UAS-myc-ago* fly that was generated with a *pUAS-myc-ago* construct in our laboratory (Additional file: Fig. S1B-D). Progeny from crosses between two *UAS-ago RNAi* lines with same *Gal4* lines showed similar phenotypes, indicating that the knockdown phenotype of *ago* is not due to off-target effects (see below).

**Fig. 1:**
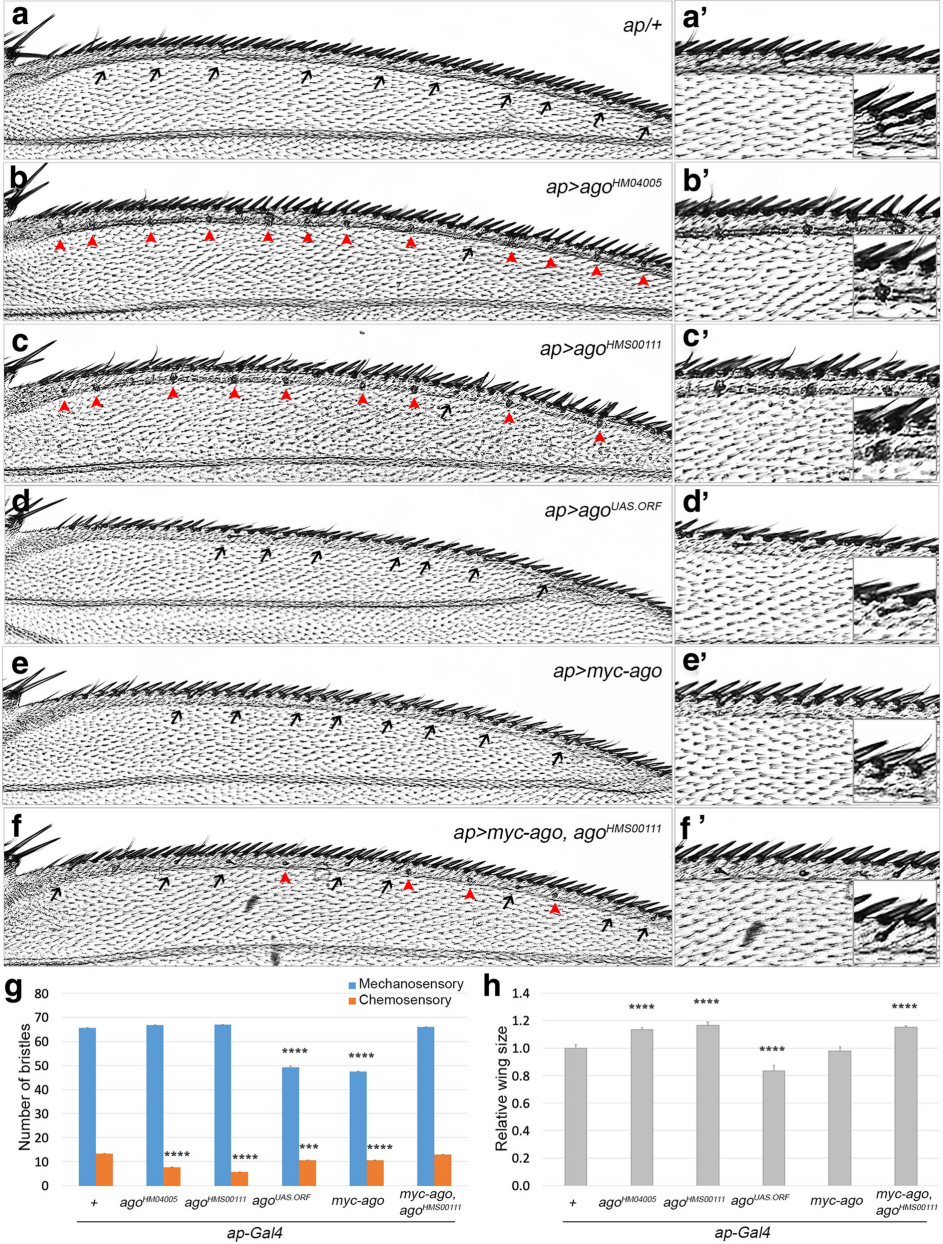
*ago* is necessary for development of chemosensory bristles at wing margin. In all figures, chemosensory bristles with shafts and without shafts are marked with black arrows and red arrowheads, respectively. **a-f** Phenotypes of chemosensory bristles in control *ap-Gal4/+* (*n* = 32, **a**), *ap > ago RNAi* (*ago*^*HM04005*^, *ago*^*HMS00111*^) (*n* = 26, **b**, and *n* = 46, **c**), *ap > ago* (*ago*^*UAS.ORF*^, *myc-ago*) (*n* = 18, **d**, and *n* = 24, **e**) and *ap > ago*^*HMS00111*^*myc-ago* (*n* = 21, **f**) wings. Images were taken by focusing on sensory bristles at dorsal anterior wing margin. Proximal to the left. Portions of the wings in (**a-f**) are magnified in (**a'-f'**) and further magnified images in insets are to show chemosensory bristles. **g** Numbers of mechanosensory and chemosensory bristles along the anterior wing margin from proximal to distal tip of the longitudinal vein 2 were counted, and the data are presented in the bar graph (***p < 0.001 and ****p < 0.0001). The blue and orange bars represent numbers of mechanosensory and chemosensory bristles, respectively. **h** Whole wing images as shown in Additional file: Fig. S2 were used to calculate wing size (****p < 0.0001)

Among *Gal4* lines tested, the two *ago RNAi* lines driven by *apterous (ap)-Gal4* driver decreased the number of chemosensory bristles to only 43% of wild-type without affecting mechanosensory bristles (Fig. 1a-c and g). Closer examination revealed, however, that shafts of the chemosensory bristles are lost but unusually enlarged sockets are still present (magnified in Fig. 1a'-c'). Unlike knockdown of *ago*, overexpression of *ago* by *ap-Gal4* reduced the number of both mechano- and chemo-sensory bristles (Fig. 1d-e and g). Expression level of Ago also affected wing size (Fig. 1h and Additional file: Fig. S2). Knockdown of *ago* by *ago*^*HM04005*^ and *ago*^*HMS00111*^ expression increased wing size by 13 and 17%, respectively, whereas *ago*^*UAS.ORF*^ and *myc-ago* expression decreased wing size by 16 and 2%, respectively (Fig. 1h). Coexpression of *ago RNAi* and *myc-ago* significantly rescued both the number of chemosensory bristles and wing size, indicating that reduction in the amount of endogenous Ago by *ago RNAi* expression is compensated by overexpression of exogenous Myc-Ago (Fig. 1f-h).

### Ago in sense organ cells is important for the formation of shafts in chemosensory bristles

**Fig. 2:**
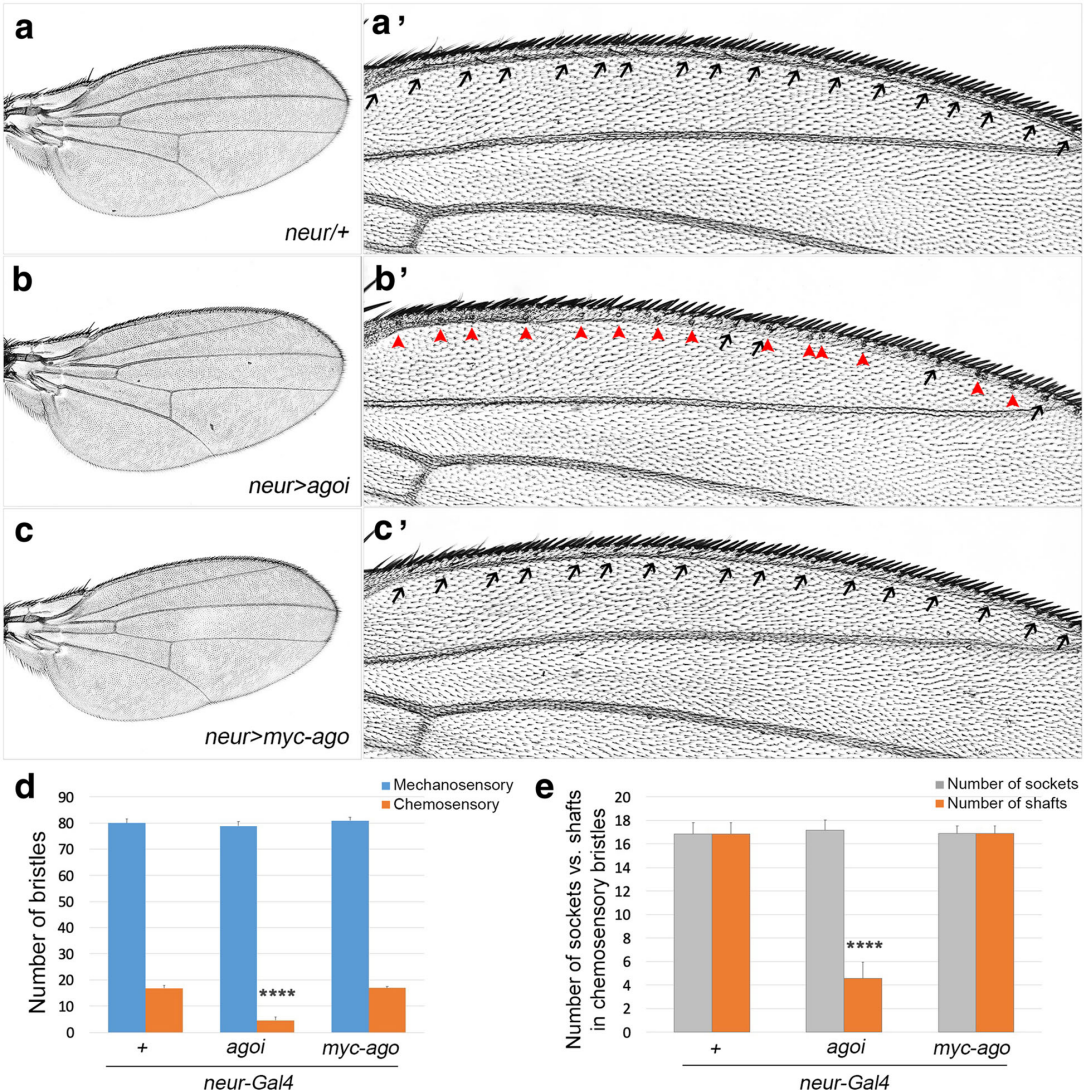
Knockdown of *ago* results in loss of shafts in chemosensory bristles. **a-c** Wings of progeny from a cross between *neur-Gal4* and *w*^*1118*^ (*n* = 24, **a**) as control, *neur > ago RNAi* (*n* = 11, **b**) and *neur > myc-ago* wings (*n* = 13, **c**). Portions of wings in (**a-c**) are magnified in (**a'-c'**). Proximal to the left. **d** Numbers of mechanosensory and chemosensory bristles of the wings described in (**a-c**) were counted. The blue and orange bars represent mechanosensory and chemosensory bristles, respectively. (****p < 0.0001). **e** Sockets with and without shafts in chemosensory bristles were counted and their numbers are presented in a bar graph with orange and grey colors, respectively. (****p < 0.0001). All flies were cultured at 25 °C

To examine the role of Ago strictly in cells of sense organ lineage, we used *neuralized (neur)-Gal4* that drives expression only in sense organ lineage (Fig. 2). Expression of *ago*^*HMS00111*^ (hereafter *UAS-ago RNAi*) by *neur-Gal4* at 25 °C did not change wing size, but decreased the number of chemosensory bristles to 27% of the control without affecting mechanosensory bristles (Fig. 2a-b',d). Overexpression of *UAS-myc-ago* affected neither wing size nor any sensory bristles (Fig. 2c,c',d). Similar to *ap > ago RNAi* wings, all defective chemosensory bristles in *neur > ago RNAi* wings had enlarged sockets without shafts (Fig. 2e). This loss of shaft phenotype was not recapitulated by expression of dMyc or Cyc E, two well-known substrates of Ago, suggesting that this phenotype is not due to accumulation of these Ago substrates (Additional file: Fig. S3). Taken together, changes in Ago level in the entire dorsal domain by *ap-Gal4* affected both wing size and sensory bristles but those in sense organ lineage by *neur-Gal4* affected only chemosensory bristles.

### *ago* and *wg* show an antagonistic relationship

**Fig. 3:**
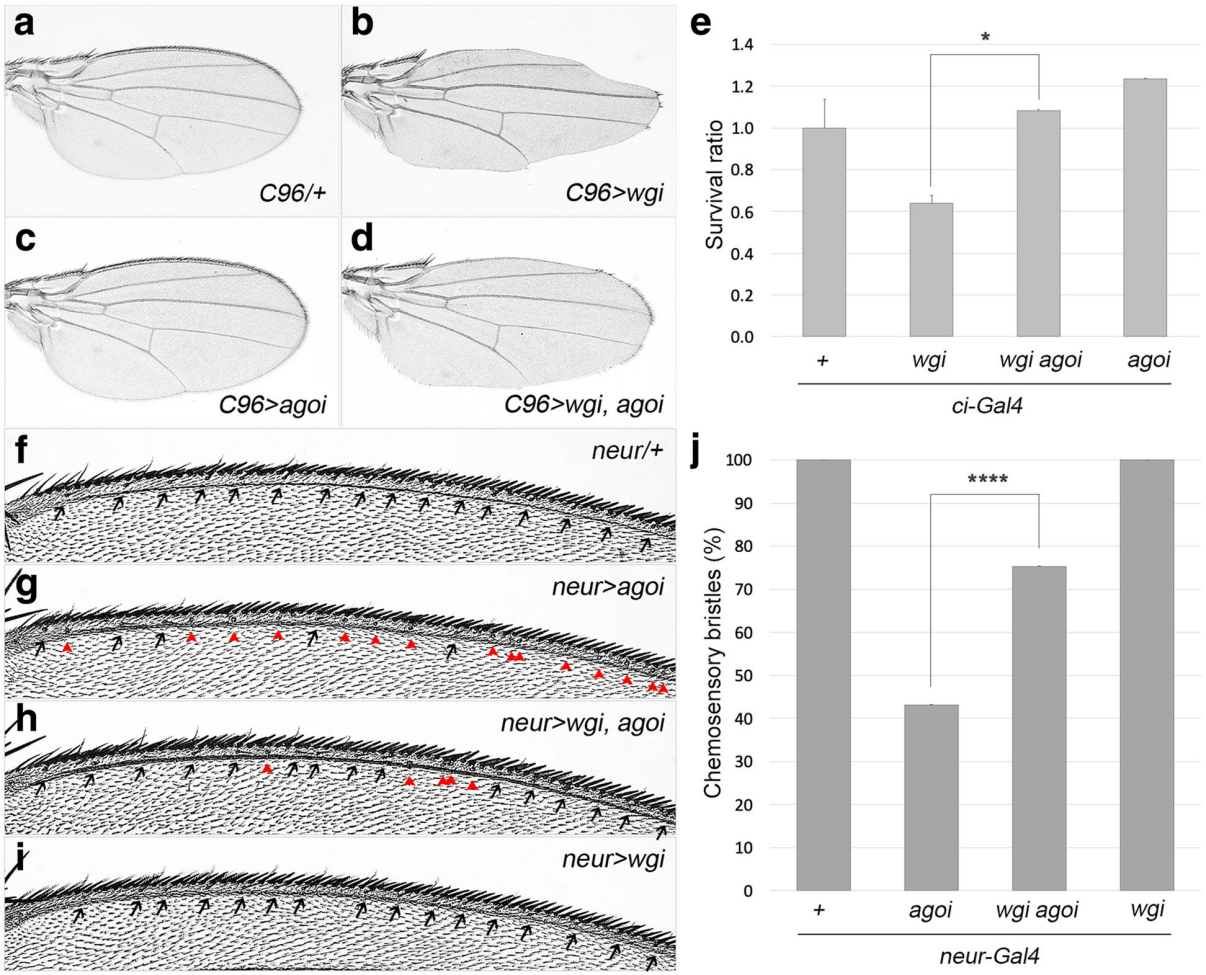
*ago* and *wg* show an antagonistic relationship. **a**-**d** Loss of wing margin by knockdown of *wg* is rescued by knockdown of *ago*. *C96-Gal4* was used to drive *wg RNAi* (**b**), *ago RNAi* (**c**) or together (**d**) at 25 °C. *C96-Gal4/+* wing as a control (**a**). **e** Lethal phenotype by knockdown of *wg* is rescued by knockdown of *ago*. That is, *ci-Gal4/CyO* females and *w*^*1118*^*, UAS-ago RNAi*, *UAS-wg RNAi* or *UAS- wg RNAi ago RNAi* males were crossed, and their progeny with *ci-Gal4,* but not *CyO,* were counted (*n* > 50 for each group). Because the control cross between *ci-Gal4/CyO* females and *w*^*1118*^ males yielded 40% *ci-Gal4/+* and 60% *+/CyO* progeny, expected factor 0.67 (40/60), was considered in calculating survival rates of progeny in experimental groups. *p < 0.05. **f**-**j** Loss of shafts in chemosensory bristles by knockdown of *ago* is rescued by knockdown of *wg*. *neur-Gal4/+* control (*n* = 19, **f)**, *neur > ago RNAi* (n = 13, **g**), *neur > ago RNAi wg RNAi* (*n* = 8, **h**) and *neur > wg RNAi* (*n* = 16, **i**) wings. Quantitative analysis of these bristles is presented in (**j**) (****p < 0.0001). Flies were cultured at 18 °C

Involvement of Ago in wing development prompted us to examine the relationship between *ago* and *wg.* We chose *C96-Gal4* that drives expression in the dorsal-ventral (DV) margin region of wing discs. We found that all *C96 > ago RNAi* wings are normal whereas all *C96 > wg RNAi* wings lack wing margin (*n* = 15 for each group, Fig. 3a-c). Interestingly, this loss of wing margin phenotype was substantially rescued by coexpression of *ago RNAi* in all wings examined (*n* = 16, Fig. 3d). Therefore, an antagonistic relationship exists between *ago* and *wg*.

We then used *ci-Gal4* to further check the relationship between *ago* and *wg*. *ci-Gal4/CyO* flies were crossed with *w*^*1118*^, *UAS-ago RNAi*, *UAS-wg RNAi* or *UAS- wg RNAi ago RNAi* flies and cultured at 18 °C, and their progenies were counted for survival rate. In all cases, flies containing *ci-Gal4* but not *CyO* were calculated (Fig. 3e). As a control, cross between *ci-Gal4/CyO* and *w*^*1118*^ yielded 40% *ci-Gal4/+* and 60% *+/CyO* progeny. Compared to control *ci-Gal4/+* progeny, *ci > ago RNAi* flies showed 24% higher survival rate whereas *ci > wg RNAi* flies showed 36% lower survival rate. Consistent with wing margin phenotype described above, the survival rate of *ci > wg RNAi* flies was increased to control level by coexpression of *ago RNAi* in *ci > wg RNAi ago RNAi* flies (Fig. 3e).

In case of chemosensory bristles, 57% of *neur > ago RNAi* wings had no shafts but only 25% of *neur > ago RNAi wg RNAi* wings had no shafts at 18 °C, indicating that knockdown of *ago* phenotype was partially rescued by knockdown of *wg* (Fig. 3f-j). Taken together, *ago* and *wg* have an antagonistic relationship.

### Amount of Arm and level of Wg signaling negatively correlate with level of Ago

**Fig. 4:**
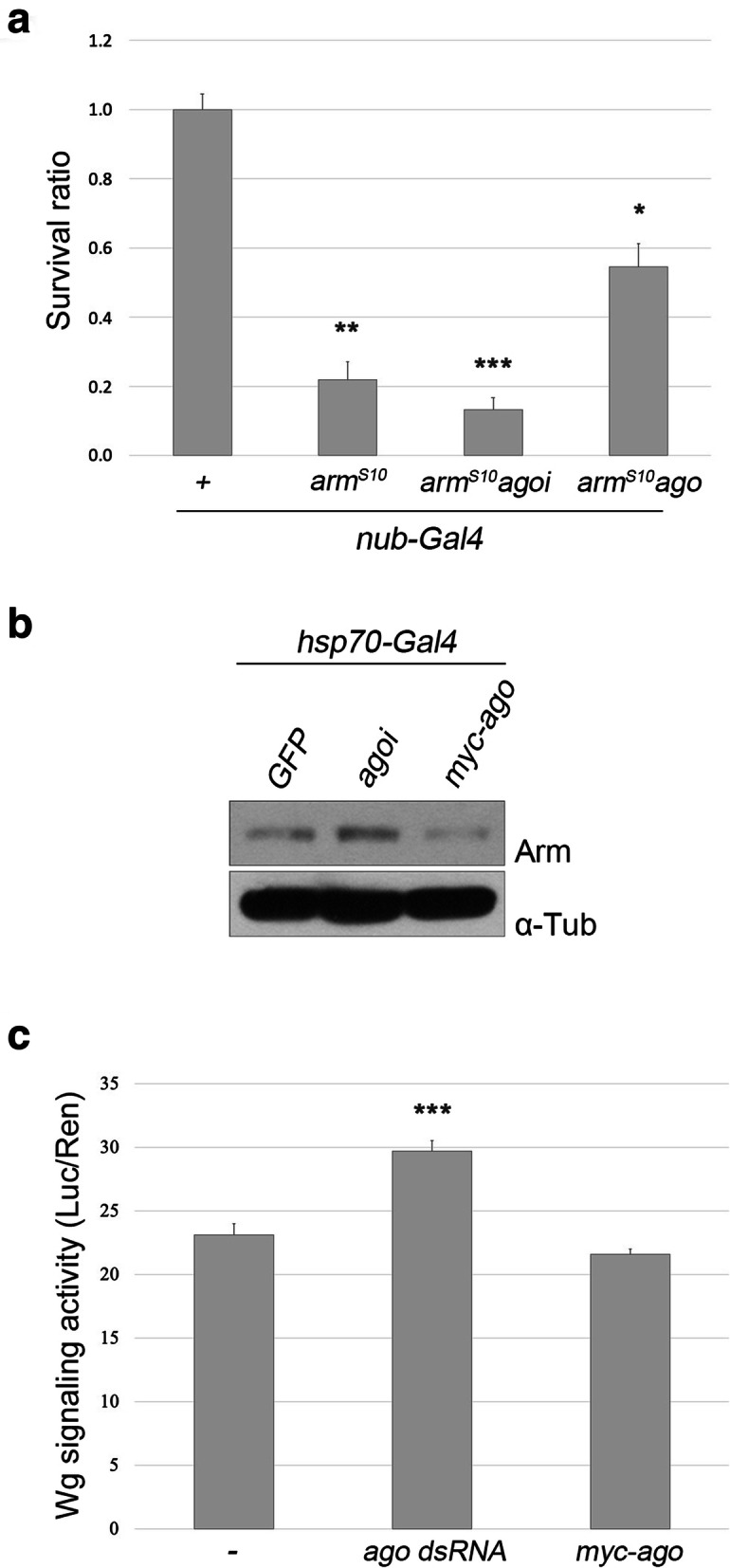
Knockdown of *ago* enhances canonical Wg signaling by increasing the level of Arm. **a** Lethality caused by overexpression of Arm^S10^ is suppressed by Ago. Only female progeny from crosses between *nub-Gal4* females and *w*^*1118*^, *UAS-arm*^*S10*^*, UAS-arm*^*S10*^*UAS-ago RNAi* or *UAS-arm*^*S10*^*UAS-ago* males were counted (*n* > 100 for each group) for survival rate since *UAS-Arm*^*S10*^ transgene is present in the first chromosome. The control cross yielded 45% female and 54% male of *nub-Gal4/+*. *p < 0.05, **p < 0.01 and ***p < 0.001. **b** Level of Arm is increased by knockdown of Ago and decreased by overexpression of Ago in cell extracts from larvae expressing GFP, *ago RNAi* and *myc-ago*. α-Tubulin as a control. **c** Knockdown of Ago enhances Wg signaling activity. S2R+ cells were transfected with *ago dsRNA* or *myc-ago,* and Wg activity was measured. Results from triplicate experiments are presented (***p < 0.001)

We then examined the relationship between Ago and Arm, a key transducer of Wg signaling because our results suggested that the loss of *ago* excessively promotes Wg signaling. When Arm^S10^, a truncated Arm protein that is constitutively active [23], was expressed by *nubbin (nub)-Gal4* at 18 °C, 78% of pupal lethality was observed (Fig. 4a). This Arm^S10^-driven lethality was slightly increased by coexpression with *ago RNAi* but significantly suppressed to 45% by coexpression with *ago*. Consistent with this, the level of endogenous Arm was increased by 19% in *hs > ago RNAi* but decreased by 11% in *hs > myc-ago* when level of Arm was compared in cell extracts from *hsp70 > GFP*, *hsp70 > ago RNAi* and *hsp70 > myc-ago* larvae that had been heat-shocked for an hour before sampling (Fig. 4b). Therefore, knockdown of *ago* increased the amount of Arm, which may enhance Wg signaling.

To further test the importance of Ago in regulation of Wg signaling, we changed the level of Ago protein by transfecting S2R+ cells with either *ago* dsRNA or *pUAS-myc-ago* construct along with WISIR vector. After 2 day-culture, these cells were cultured for 20 h with Wg that had been obtained from the conditioned media of S2 *tub-wg* cell culture. Finally, activity of Wg signaling was measured by TOP Flash assay with the WISIR vector (Wingless Signaling Reporter) [24]. We found that *ago* dsRNA-treated cells show 30% higher Wg activity while MYC-Ago-expressing cells show 9% lower Wg activity than control cells (Fig. 4c). These data are consistent with the findings on the negative interaction between *ago* and *wg*.

### Knockdown of *ago* increases level of extracellular Wg secreted from Wg-producing cells

**Fig. 5:**
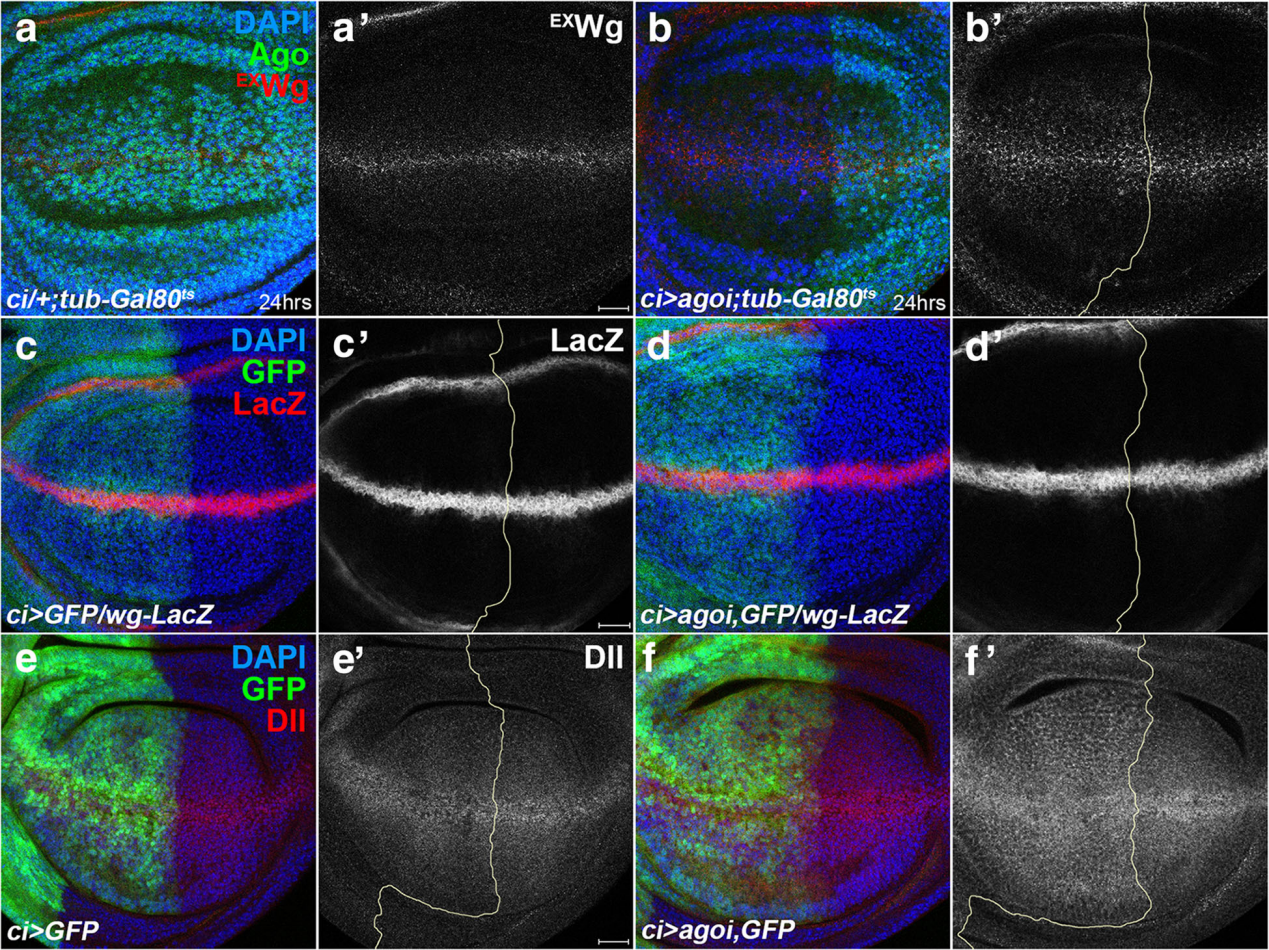
Knockdown of Ago increases level of extracellular Wg post-transcriptionally. Yellow lines delimit the anterior and posterior compartments. **a-b** Knockdown of Ago increases level of extracellular Wg. Larval progeny from crosses between *w*^*1118*^ (**a**) or *UAS-ago RNAi* (**b**) with *ci-Gal4; tub-Gal80*^*ts*^ flies were used to obtain wing discs. Knockdown of Ago by *ago RNAi* was confirmed with anti-Ago antibody. **c-d** Knockdown of Ago did not change *wg*-LacZ pattern. *ci > GFP/wg-lacZ* control (**c**) and *ci > GFP, ago RNAi/wg-lacZ* (**d**) wing discs show no difference in *wg*-LacZ pattern. **e-f** The level of Dll is enhanced by knockdown of Ago*.* Scale bar, 20 μm.

We have shown that knockdown of *wg* by *C96-Gal4* is rescued by knockdown of *ago* (Fig. 3a-d)*. C96-Gal4* drives expression in cells at the DV midline that includes Wg-producing cells (Additional file: Fig. S4). Thus, we checked whether knockdown of *ago* induces any changes in Wg-producing cells. To avoid any developmental defects by prolonged knockdown of *ago, ago RNAi* was expressed only for 24 h using the inducible *Gal4-Gal80*^*ts*^ system [25]. When *ago RNAi* was expressed by *ci-Gal4* for 24 h, the level of extracellular Wg was increased especially in the pouch region of the anterior compartment compared to control discs (*n* = 7, Fig. 5a,b). This increase was not due to the increase by *wg* transcription since LacZ level by *wg-lacZ* expression was not increased by the loss of *ago* (*n* = 10, Fig. 5c,d). Additionally, the level of Dll in *ci > ago RNAi* wing discs was increased in the anterior region*,* consistent with the higher level of extracellular Wg in the pouch region (*n* = 11, Fig. 5e,f). Therefore, we conclude that knockdown of Ago increases the level of extracellular Wg by the post-transcriptional mechanism.

If Ago regulates the level of extracellular Wg, knockdown of Ago in the wing pouch might affect the shape of Wg gradient. To test this, the intensity of extracellular Wg was quantitatively measured in *ap > ago RNAi* wing discs (*n* = 5, Additional file: Fig. S5). When *ago RNAi* was transiently expressed for 24 h by *ap-Gal4*, the gradient of extracellular Wg in the dorsal compartment became less steep than control discs (Additional file: Fig. S5b). This result supports a new role of Ago in regulating the level of extracellular Wg.

### Amount of secreted Wg negatively correlates with that of Ago in S2 cells

**Fig. 6:**
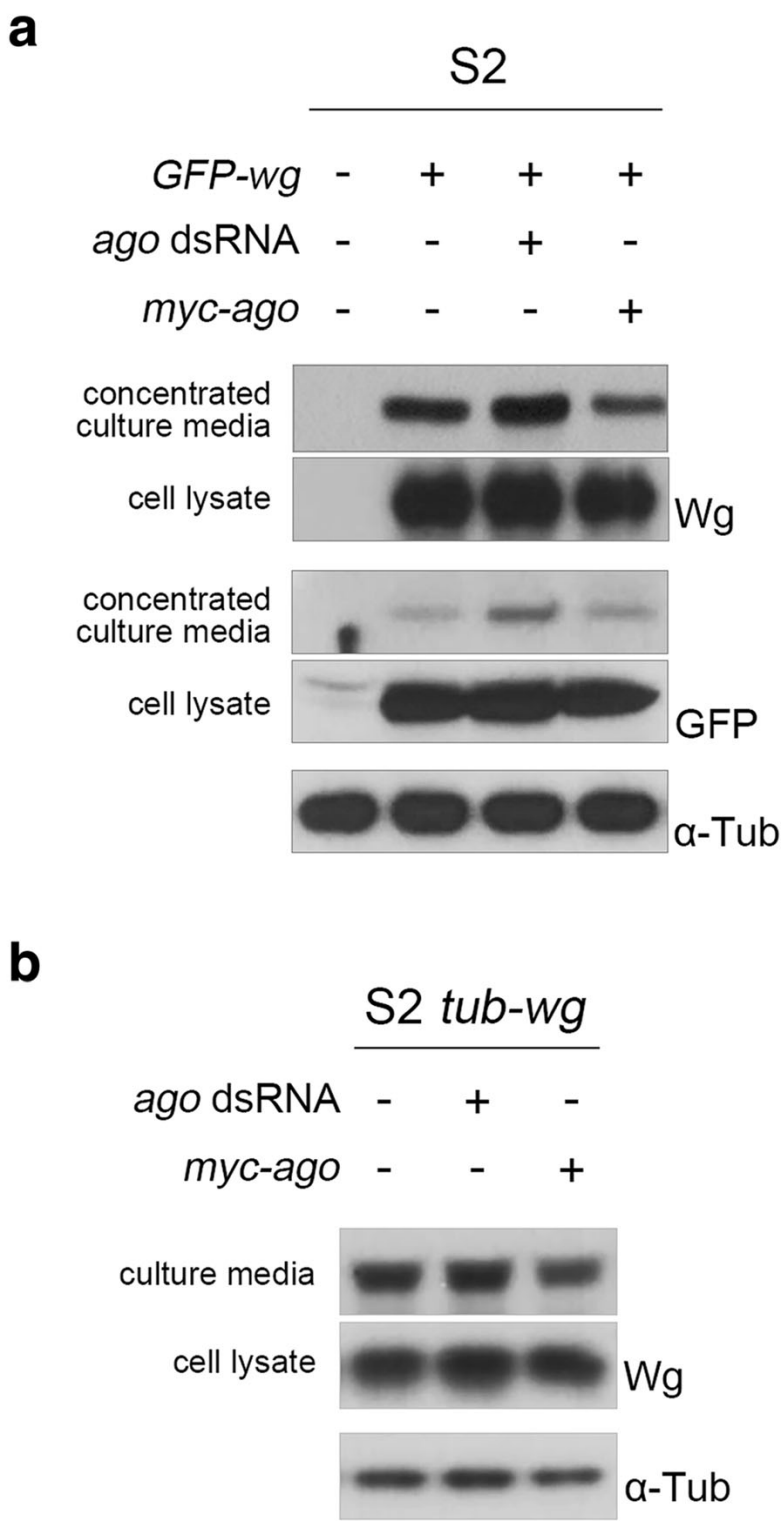
Overexpression of Ago decreases Wg secretion, and loss of Ago increases Wg secretion in vitro**.a** Amount of Wg in conditioned media is increased by loss of Ago and decreased by gain of Ago in S2 cells. S2 cells are co-transfected with *GFP-wg* in combination with either *ago* dsRNA or *myc-ago*. Extracellular Wg in culture media and intracellular Wg in cell extracts were detected with both anti-GFP and anti-Wg antibodies. α-Tubulin was used as an internal control. **b** Level of extracellular Wg was decreased by *myc-ago* expression in S2 *tub-wg* cells

To further examine the role of Ago in regulating the level of extracellular Wg, we took a biochemical approach with fly S2 cell culture. S2 cells do not express Wg endogenously [26]; therefore, cells were transfected with *GFP-wg* in combination with either *ago* dsRNA or *myc-ago* (Fig. 6a). After 3-day culture, extracellular Wg was obtained by concentrating conditioned media after removal of dead cells and debris by centrifugation. When GFP-Wg and *ago* dsRNA were co-expressed, we found that the level of secreted Wg detected by anti-GFP antibody or anti-Wg antibody was increased by 33 and 10%, respectively. In contrast, cells co-expressing MYC-Ago and GFP-Wg secreted 20% less GFP-Wg detected by anti-Wg antibody. When using S2 *tub-wg* cell line that constitutively expresses Wg, Wg was secreted 14% more by *ago* knockdown and 19% less by *myc-ago* overexpression (Fig. 6b). These data established that Ago decreases the level of extracellular Wg secreted from Wg-producing cells.

### High level of Ago causes accumulation of Wg in ER

**Fig. 7:**
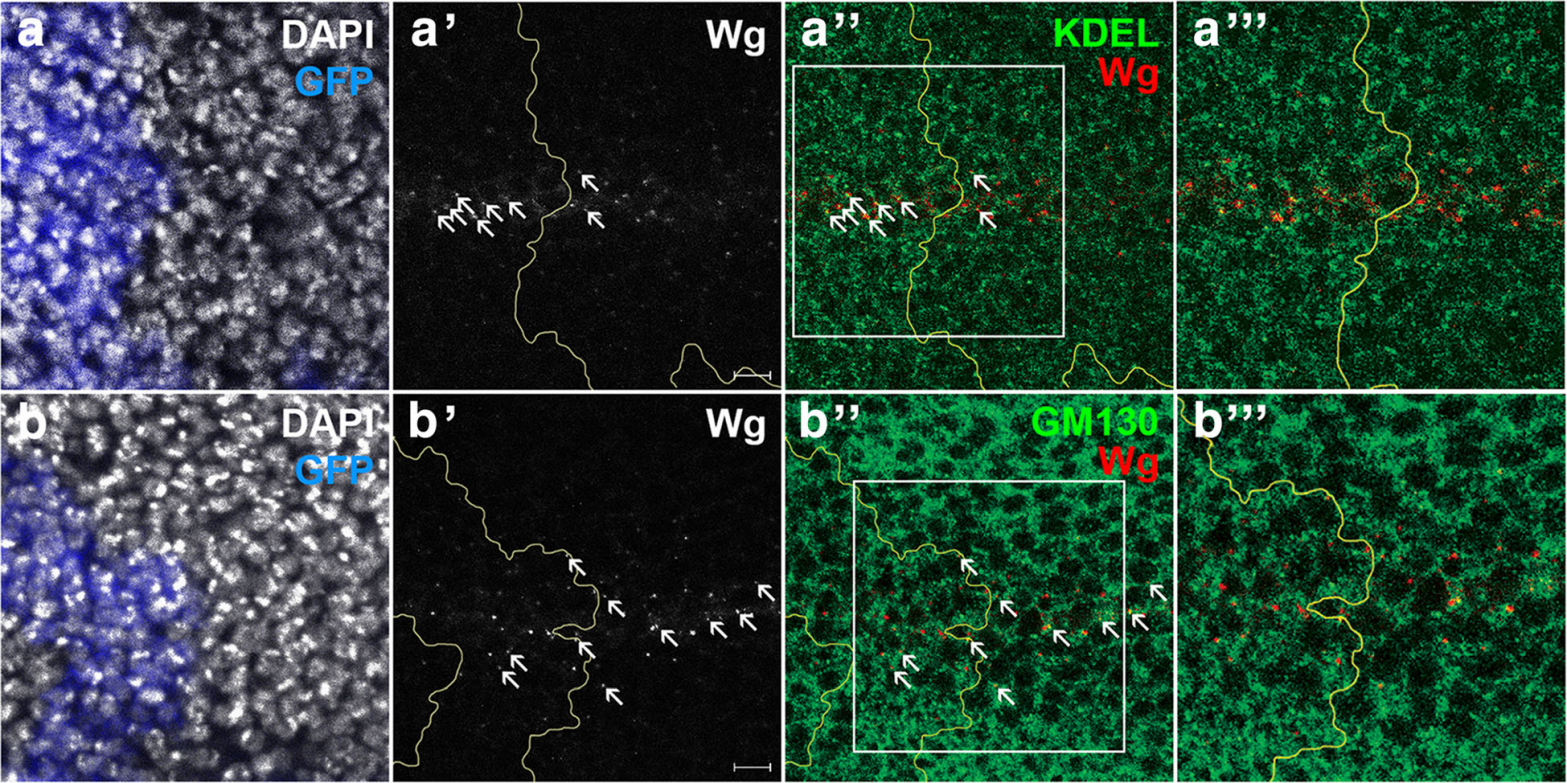
Overexpression of Ago increases the level of Wg in ER. Clones that overexpress Ago were generated in wing discs outlined in yellow. Endogenous intracellular Wg was detected with anti-Wg, and the clones were marked with GFP. Boxed area in (**a''**) and (**b''**) are magnified in (**a'''**) and (**b'''**). **a** More co-localization of Wg and ER marker KDEL is observed in an Ago-expressing clone than neighboring cells (arrows). **b** Less co-localization of Wg and Golgi marker GM130 is seen in an Ago-expressing clone than neighboring cells (arrows). Scale bars, 5 μm

To understand how Ago inhibits secretion of Wg, we checked whether the level of Wg is increased in any subcellular compartments such as ER or Golgi of the Ago-expressing cells in the DV midline since this region is marked by high expression level of endogenous Wg. We generated Ago-overexpressing clones and tested with anti-KDEL and anti-GM130 antibodies that visualize ER and Golgi apparatus, respectively. We found that Wg is more frequently co-localized with KDEL than GM130 in large clones encompassing the DV midline (*n* = 10, Fig. 7). These results suggest that Ago prevents transport of Wg from ER to Golgi. Although further analysis is required to understand the underlying mechanism of Ago in Wg transport, higher level of Wg in ER by Ago is consistent with the lower level of secreted Wg by Ago overexpression in S2 cell culture.

## Discussion

We have shown here that loss of Ago increases the level of Arm in Wg-responding cells, and it also increases the level of extracellular Wg secreted from Wg-producing cells. Therefore, Ago functions in both cell types to additively reduce activity of Wg signaling. These data are consistent with the antagonistic relationship between *ago* and *wg* in phenotypic analyses on wings and chemosensory bristles (Fig. 3). Wing notching phenotype by loss of *wg* was rescued by loss of *ago*, while lack of shafts in chemosensory bristles by loss of *ago* was rescued by loss of *wg.* Similar antagonistic relation between the two genes has been reported in both flies and mammalian cells. Apoptosis in eye imaginal discs of an *ago* mutant is suppressed by loss of *wg*, and ubiquitination of Forkhead box protein M1 (FoxM1) by FBW7 is inhibited by Wnt [27, 28]. Taken together, we conclude that Ago decreases activity of Wg signaling by acting in both Wg-producing and Wg-responding cells, and Ago plays a previously unidentified role in sensory organ development.

Sensory organ precursor (SOP) cells asymmetrically divide to form two external cells, socket and shaft, and two inner cells, neuron and sheath [29]. Socket and shaft cells are two daughter cells generated from a pIIa SOP cell during late 3rd instar to pupal stage [30]. Based on the absence of shafts and enlarged sockets by knockdown of *ago*, we propose that Ago is involved in the specification of shaft and socket (Fig. 2e). It is plausible that Ago is essential for the formation of shafts, and both daughter cells from the pIIa cell become socket cells in loss of *ago* condition. Involvement of E3 ubiquitin ligases such as Neur, Mindbomb (Mib) and *Drosophila* inhibitor of apoptosis1 (Diap1) in sensory bristle formation has been previously reported. For instance, Neur and Mib act prior to SOP formation for non-neuronal fate by regulating Notch signaling activity [31]. On the other hand, Diap1 is dynamically expressed in SOP cells and differentiating sensory organ cells during early pupal stage and is specifically degraded in shaft cells in order to regulate shaft cell morphogenesis [32]. It is interesting to note that Ago/FBW7 decreases whereas Mib1 promotes Wnt signaling [33]. Regulation of Wg signaling by these E3 ubiquitin ligases seems to be important for the formation of sensory organs in flies.

The level of Arm was increased by knockdown of Ago but decreased by gain of Ago (Fig. 4b). It has been shown that FoxM1 phosphorylated by Glycogen synthase kinase 3 (GSK3)/Shaggy is ubiquitinated by FBW7 and degraded when Wnt signaling is inactive. As a result, the level of nuclear FoxM1 that recruits β-cat becomes low, and transcription of Wnt effector genes are not induced. The Level of β-cat is increased by loss of Ago in human pancreatic cancer cells but there is no evidence yet that β-cat is a substrate of FBW7. Therefore, it will be interesting to find out if modulation of Arm level by Ago is via Forkhead box subtype O (FoxO) in flies or if Arm is a direct substrate of Ago.

The level of secreted extracellular Wg was increased by knockdown of Ago and decreased by overexpression of Ago in S2 cell culture (Fig. 6). Ago inhibits Wg secretion from producing cells, and Wg seemed to be held in ER in these cells (Fig. 7). Ago may degrade unknown protein(s) that enhances transport of Wg from ER to Golgi. Alternatively, high level of Ago may affect sorting signals important for Wnt transport [34]. For instance, the amount of Fz in the plasma membrane is regulated by internalization through balanced ubiquitination and deubiquitination [35], and LRP6, a mammalian homolog of Arr, cycles between mono-ubiquitination and deubiquitination in ER until proper protein folding is completed before ER exit [36]. Further detailed analysis is required for understanding how Ago plays as a regulator for Wg trafficking and signal transduction.

## Conclusions

Our data established that Ago is essential for wing growth and formation of chemosensory bristles. Gain of Ago decreases the level of Arm and knockdown of Ago increases the level of Arm and extracellular Wg in both wing discs and S2 cells. Meanwhile, overexpression of Ago decreases the level of secreted Wg in S2 cell culture, which may be due to accumulation of Wg in ER instead of transport to Golgi. We demonstrated that antagonistic relationship between *ago* and *wg* is essential for wing growth and differentiation of chemosensory organs at wing margin.

## Methods

### Fly strains

Two *ago RNAi* lines, #31501 and #34802, were obtained from Bloomington *Drosophila* stock center (BDSC). The targeting regions for BDSC#31501 and #34802 are 2504-2913th and 5538-5558th nucleotide, respectively (Additional file: Fig. S1 a). Two *ago* overexpression fly lines used are: FlyORF library #F001828 and line generated with *pUAS-myc-ago* construct from our laboratory. To make *pUAS-myc-ago*, an *ago* cDNA clone (*Drosophila* Genomics Resource Center clone #LD30271) was sequenced and cloned into *pUAST-myc*.

All *Gal4* lines were obtained from BDSC except *ci-Gal4* (gift from R. A. Holmgren). *wg-LacZ* [37], *tub-Gal80*^*ts*^ (BDSC#7017), *UAS-wg RNAi* (NIG#4889R-3), *UAS-arm*^*S10*^ (BDSC#4782), *UAS-cyc E* (BDSC#4781), *UAS-dmyc* (BDSC#9674), *UAS-nuclear lacZ* (BDSC#3955) and *UAS-GFP* (BDSC#1522) lines were also used. All cultures were carried out at either 18 °C or 25 °C. For conditional induction of *Gal4*, flies with *tub-Gal80*^*ts*^ were incubated at 18 °C until induction at 29 °C.

### Wing analysis

Adult wings were dissected from 2 to 3 days-old female adults. Wings were immersed in absolute ethanol and then transferred to mounting medium (80% glycerol in 1X PBS) [38]. Treated wings were then mounted on a slide glass with mounting medium. Wing images were obtained using microscopy under bright field with 40X or 100X magnification and used to count bristles.

Statistical analysis was performed to compare different genotypes to a wild-type control within the experimental groups. Image J program was used to measure the size of adult wings. Data are presented with the standard error of mean (SEM) for multiple batches or the standard deviation (SD) for a single batch of experiments. For statistical significance, t-test of Microsoft Excel was used.

### Cell culture and transfection

S2 (stock #6), S2R+ (stock #150) and S2-*tub-wg* (stock #165) were obtained from *Drosophila* Genomics Resource Center (DGRC). S2 cells were maintained with 10% artificial serum (Sigma-Aldrich) in M3 media (Sigma-Aldrich). S2R+ and S2-*tub-wg* cells were cultured in M3 + BPYE containing 10% fetal bovine serum (FBS, Hyclone). 125 μg/ml of hygromycin B (Invitrogen) was added to S2 *tub-wg* culture, and Cellfectin II (Invitrogen) was used for transfection. For induction of *pUAS* constructs, *pActin-Gal4* DNA was co-transfected with either *pUAS-myc-ago* or *pUAS-GFP-wg*.

### Double stranded RNA (dsRNA) synthesis

Double-stranded RNA to reduce the level of *ago* mRNA (*ago* dsRNA) in S2 cells was synthesized in vitro using MEGAscript RNAi Kit (Ambion). The LD30271 cDNA was used as a template for PCR using forward 5′-AGCAGTGAATCCGTGAC and reverse 5′-CCAGCAAGAACTCATCGCTCA primers conjugated downstream of T7 promoter sequences as written in the manufacturer’s instructions. Cells were treated with 35 nM *ago* dsRNA for 2 to 3 days until harvest.

### TOPFlash assay

S2R+ cells were transfected with either *pUAS-myc-ago* or *ago* dsRNA in the presence of WISIR (gift from J. P. Vincent). After 2 days, transfected S2R+ cells were treated with exogenous Wg that had been prepared by concentrating S2 *tub-wg* cultured media with Amicon centrifugal filter. After 20 h incubation, cells were lysed with 1X passive lysis buffer, and LAR II and STOP&Glo reagents were added (Dual Luciferase Reporter Assay System, Promega). Both firefly luciferase (Luc) and Renilla luciferase (Ren) levels were measured three times. The mean of Luc/Ren ratios were plotted in a bar graph. Error bars indicate standard deviation, and *p* value was assessed for statistical significance.

### Immunoblot analysis

Third instar larvae or S2 cells were lysed with lysis buffer and boiled with sample loading buffer at 95 °C for 10 mins [39]. Prepared samples were loaded on 7.5% SDS-PAGE gel, and protein bands in the gel were transferred to nitrocellulose membrane by wet method. Membranes were blocked with 5% dry milk in TBS-T (140 mM NaCl; 3 mM KCl; 25 mM Tris pH 7.4; 0.1% Tween-20) at room temperature (RT) for 1 h. Membranes were sequentially probed in primary antibody and HRP-conjugated secondary antibody solution diluted with 2% dry milk in TBS-T. After washing, membranes were developed with WESTSAVE-Gold reagent (AbFrontier).

Primary antibodies used are: concentrated anti-Wg (mouse, 1:2000, Developmental Studies Hybridoma Bank (DSHB)), anti-GFP (rabbit, 1:10,000, abcam), anti-αTub (mouse, 1:5000, Sigma-Aldrich), anti-MYC (rabbit, 1:5000, abcam) and anti-Ago (guinea pig, 1:5000, gift from K. H. Moberg). Secondary antibodies used are: HRP-conjugated anti-mouse antibody (1:10,000, Jackson Laboratory), HRP-conjugated anti-rabbit antibody (1:10,000, Jackson Laboratory) and HRP-conjugated anti-guinea pig antibody (1:5000, Jackson Laboratory).

### Immunohistochemistry

Wing discs were dissected from third instar larvae and stained as described [40, 41]. After fixing, tissues were immersed in blocking solution for 1 h at RT. The tissues were then incubated at 4 °C overnight sequentially in the primary and secondary antibodies diluted in washing buffer. Lastly, tissues were stained with DAPI. Vectashield (Vector Laboratories, USA) was used for mounting tissues. The following primary antibodies were used for staining: anti-Wg (mouse, 1:100, DSHB), anti-MYC (rabbit, 1:500, abcam), anti-LacZ (chicken, 1:100, abcam), anti-Dll (goat, 1:100, Santa Cruz) and anti-Ago (guinea pig, 1:500, gift from K. H. Moberg). Fluorescent images were acquired using Zeiss confocal microscope LSM 710.

### Extracellular Wg staining

For extracellular staining of Wg, larvae were dissected and probed as described [26]. After incubation in concentrated anti-Wg (1:100) primary antibody solution, wing discs were fixed with 4% paraformaldehyde in 1X PBS at RT for 1 h. After washing twice with 1X PBS, samples were immersed in blocking solution without detergent for 1 h at RT. Then the samples were incubated with secondary antibody diluted in detergent-free washing buffer at 4 °C overnight. These samples were washed with detergent-free washing buffer, and then permeabilized in blocking solution containing detergent used for conventional staining. The samples were then stained as described above.

### Clonal analysis

To generate *ago* overexpression clones, *hsp70-FLP* (BDSC#1929) and *UAS-ago*^*UAS.ORF*^ (FlyORF#F001828) were recombined, and *hsp70-FLP UAS-ago* flies were generated. These flies were crossed with *Ay-Gal4,UAS-GFP/CyO* (BDSC#4411) and cultured at 18 °C for 5–6 days. Larvae were then shifted to 37 °C for an hour, moved to RT and cultured for 4–5 days. Third instar larvae were then selected and stained by following the conventional staining method described above. The following antibodies were used: Anti-Wg (mouse, 1:100, DSHB), anti-KDEL (rabbit, 1:200, abcam) and anti-GM130 (rabbit, 1:200, abcam).

## Supplementary information

**Additional file 1: Figure S1.** Validation of *myc-ago* and *ago* dsRNA constructs. **a** Target regions of HM04005 and HMS00111 are marked with red bars on corresponding regions in Ago protein. **b** S2 cells transfected with *myc-ago* expressed MYC-Ago, and S2 cells cotransfected with both *myc-ago* and *ago* dsRNA did not express MYC-Ago. Endogenous Ago in S2 cells is undetectable as shown in the first lane. **c** The level of MYC-Ago was increased as culture time after heatshock at 37^o^C from 1 to 6 hours in *hsp70>myc-ago* larval extract*.***d** MYC-Ago was also detected by both anti-MYC and anti-Ago antibodies in wing discs. Scale bar, 20 μm.

**Additional file 2: Figure S2.** Loss or gain of *Ago* affects wing size. *ap-Gal4* was crossed with *w*^*1118*^ (n=28, **a**), *UAS-ago*^*HM04005*^ (n=19, **b**), *UAS-ago*^*HMS00111*^ (n=18, **c**), *UAS-ago*^*UAS.ORF*^ (n=17, **d**), *UAS-myc-ago* (n=20, **e**) and *UAS-myc-ago UAS-ago*^*HMS00111*^ (n=20, **f**) at 25ºC. These whole wing images were used to calculate the wing size in Fig. 1h.

**Additional file 3: Figure S3.** Loss of shafts in chemosensory bristles by knockdown of *ago* is independent of two Ago substrates, Cyc E and dMyc. **a-c***neur-Gal4* was crossed with *UAS-ago RNAi*, *UAS-cyc E* or *UAS-dmyc* and cultured at 18ºC. Knockdown of *ago* induced loss of shafts in chemosensory bristles (**a**), but knockdown of *cyc E* (n=12, **b**) or *dmyc* (n=10, **c**) did not.

**Additional file 4: Figure S4.** Wg-expressing cells are a subpopulation of *C96-Gal4*^+^ cells in DV midline. **a-b** Pattern of nuclear LacZ representing *C96-Gal4*^*+*^ cells (**a'**) and *wg*-LacZ^+^ cells (**b'**). **c** Wing discs expressing GFP driven by *C96-Gal4* and LacZ by *wg-LacZ* reporter. Scale bar, 20 μm.

**Additional file 5: Figure S5.** Knockdown of Ago reduces the steepness of Wg gradient. *ago RNAi* was induced for 24 h by *ap-Gal4; tub-Gal80ts*. **a** Wing discs were obtained immediately after Gal4 induction and stained for extracellular Wg. A representative image was presented (n=5). The composite image in (**a**) was individually shown in (**a'-a'''**). Yellow line distinguishes the DV midline based on anti-Ago (**a'''**). **b** The graph shows fluorescent intensity of extracellular Wg (green line) and Ago (red line) crossing DV midline marked in (**a**).

## Data Availability

The datasets used or analysed during the current study are available from the corresponding author on reasonable request.

## Abbreviations

Ago: Archipelago
FBW7: F-box and WD repeat domain-containing 7
β-Cat: beta-catenin
Wg: Wingless
Arm: Armadillo
Dll: Distal-less
ER: Endoplasmic reticulum
Trh: Trachealess
Sima: Similar
Cyc E: Cyclin E
Fz: Frizzled
Arr: Arrow
*ap*: *apterous*
*neur*: *neralized*
DV: dorsal-ventral
*nub*: *nubbin*
WISIR: Wingless signaling reporter
FoxM1: Forkhead box protein M1
SOP: Sensory organ precursor
Mib: Mindbomb
Diap1: Death-associated inhibitor of apoptosis1
GSK3: Glycogen synthase kinase3
FoxO: Forkhead box subtype O
LRP6: Low-density lipoprotein (LDL)-related protein 6
