# Supplementary figures and images for: Wingless and Archipelago, a fly E3 ubiquitin ligase and a homolog of human tumor suppressor FBW7, show an antagonistic relationship in wing development
Source: BMC Dev Biol. 2020 Jun 29;20:14. doi: 10.1186/s12861-020-00217-1 (PMC7322864; doi:10.1186/s12861-020-00217-1)

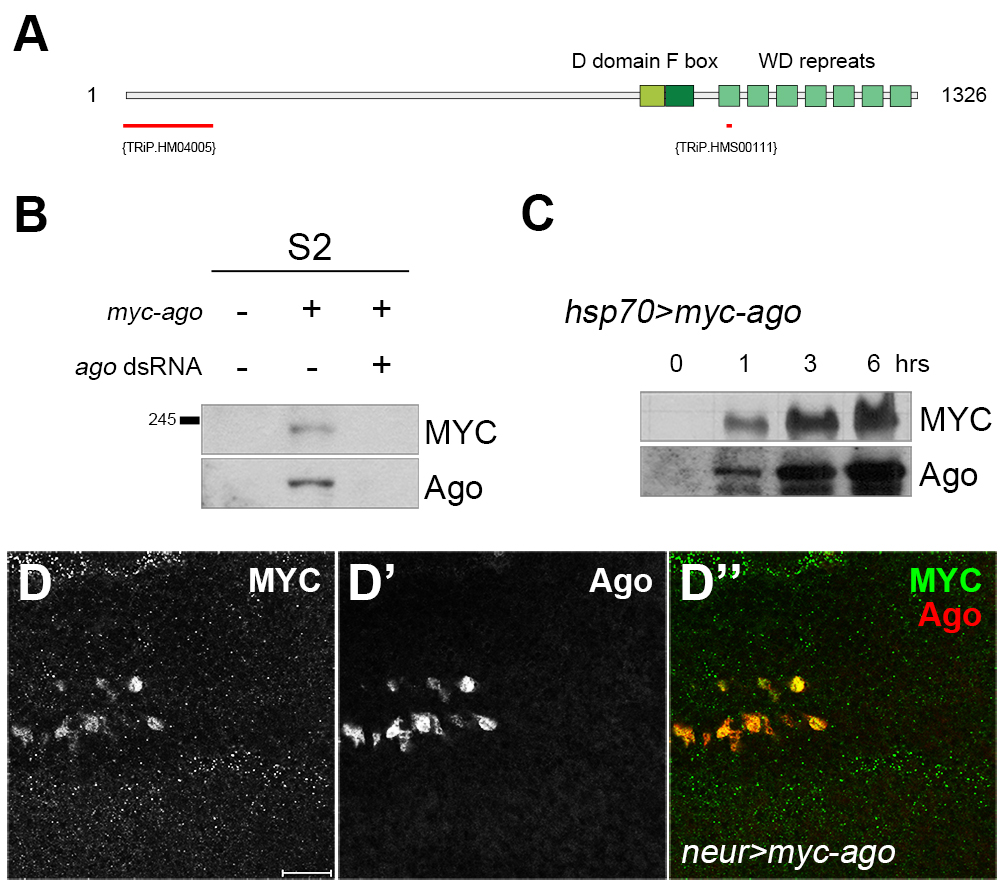

Supplement: Supplementary file 1 — Additional file 1: Figure S1. Validation of myc-ago and ago dsRNA constructs. a Target regions of HM04005 and HMS00111 are marked with red bars on corresponding regions in Ago protein. b S2 cells transfected with myc-ago expressed MYC-Ago, and S2 cells cotransfected with both myc-ago and ago dsRNA did not express MYC-Ago. Endogenous Ago in S2 cells is undetectable as shown in the first lane. c The level of MYC-Ago was increased as culture time after heatshock at 37oC from 1 to 6 hours in hsp70>myc-ago larval extract.d MYC-Ago was also detected by both anti-MYC and anti-Ago antibodies in wing discs. Scale bar, 20 μm. [file 12861_2020_217_MOESM1_ESM.jpg]

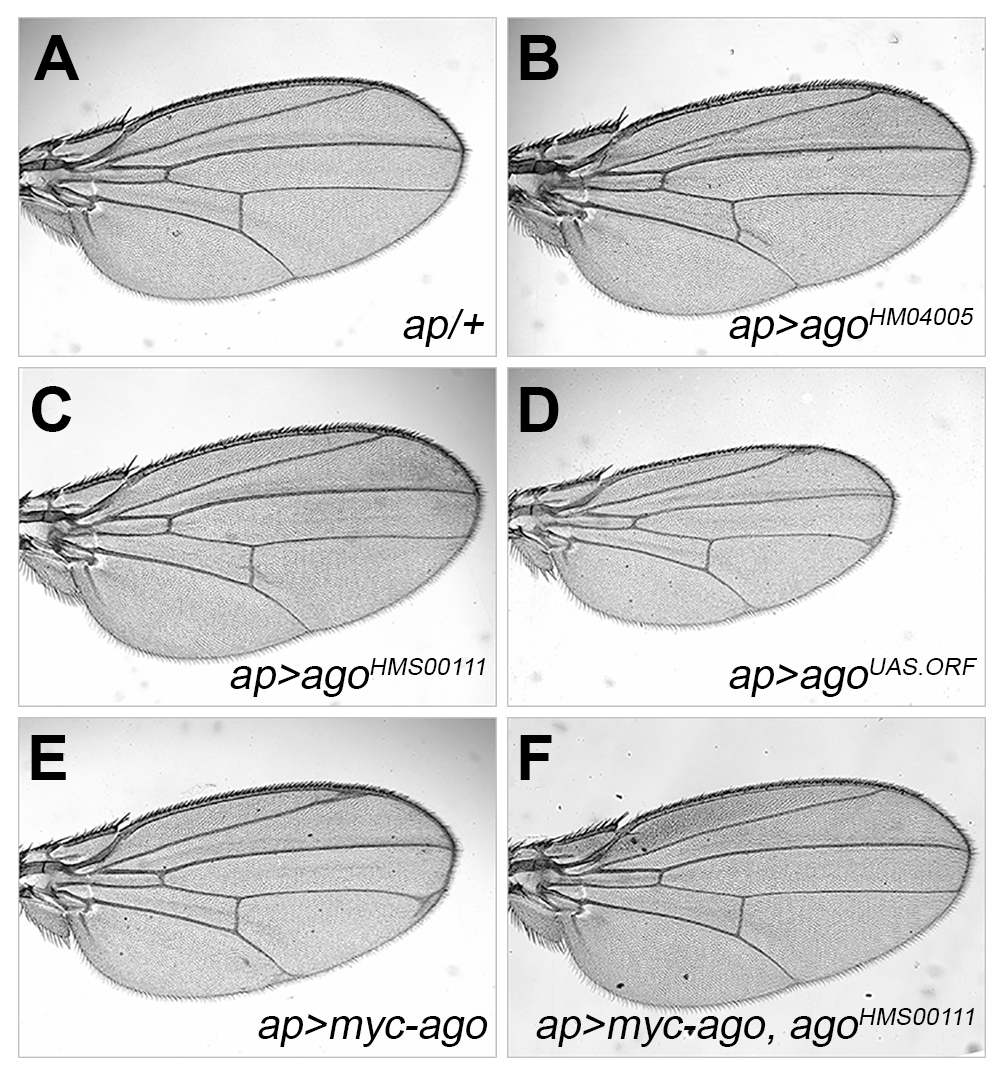

Supplement: Supplementary file 2 — Additional file 2: Figure S2. Loss or gain of Ago affects wing size. ap-Gal4 was crossed with w1118 (n=28, a), UAS-agoHM04005 (n=19, b), UAS-agoHMS00111 (n=18, c), UAS-agoUAS.ORF (n=17, d), UAS-myc-ago (n=20, e) and UAS-myc-ago UAS-agoHMS00111 (n=20, f) at 25ºC. These whole wing images were used to calculate the wing size in Fig. 1h. [file 12861_2020_217_MOESM2_ESM.jpg]

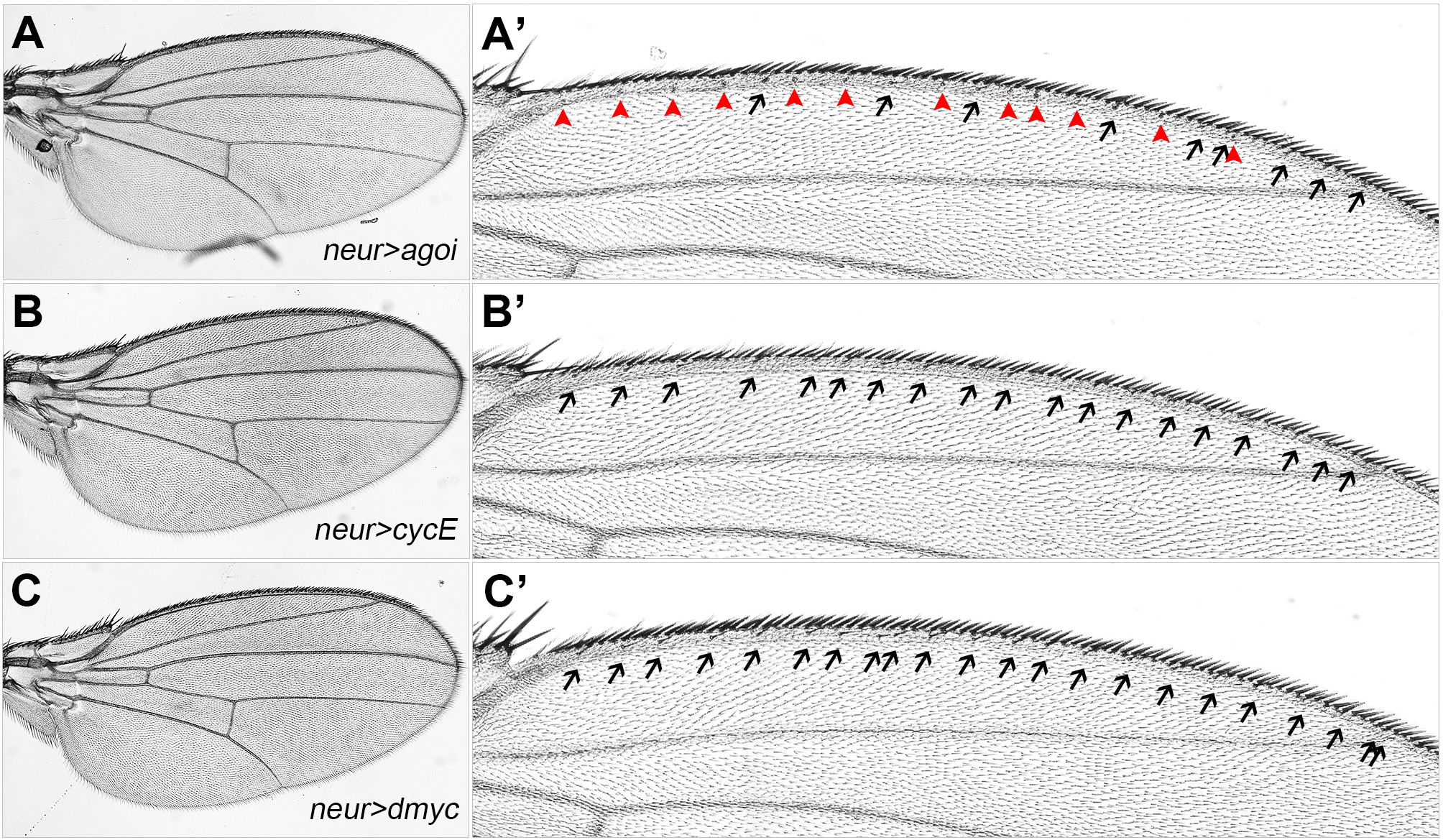

Supplement: Supplementary file 3 — Additional file 3: Figure S3. Loss of shafts in chemosensory bristles by knockdown of ago is independent of two Ago substrates, Cyc E and dMyc. a-cneur-Gal4 was crossed with UAS-ago RNAi, UAS-cyc E or UAS-dmyc and cultured at 18ºC. Knockdown of ago induced loss of shafts in chemosensory bristles (a), but knockdown of cyc E (n=12, b) or dmyc (n=10, c) did not. [file 12861_2020_217_MOESM3_ESM.jpg]

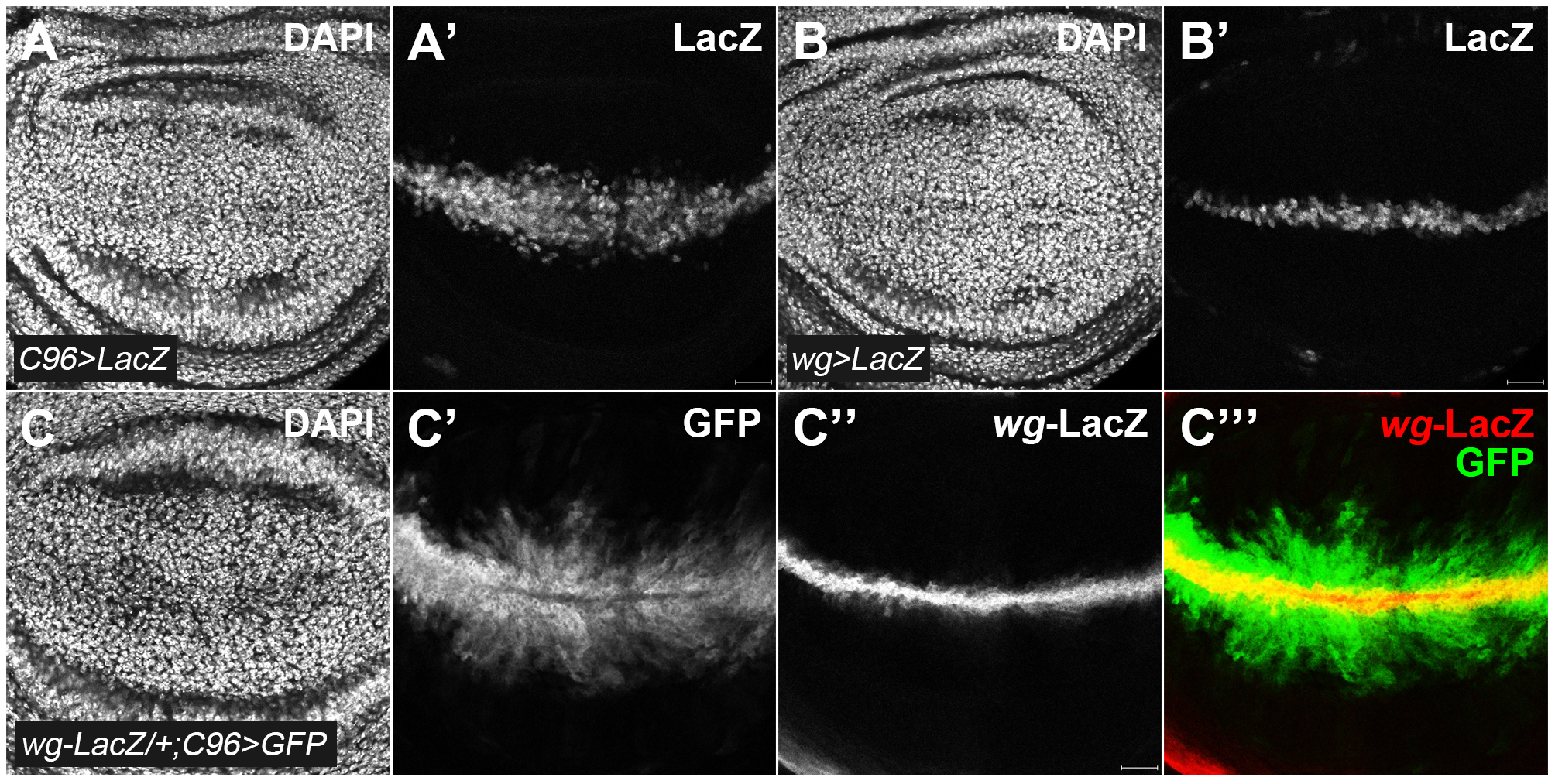

Supplement: Supplementary file 4 — Additional file 4: Figure S4. Wg-expressing cells are a subpopulation of C96-Gal4+ cells in DV midline. a-b Pattern of nuclear LacZ representing C96-Gal4+ cells (a') and wg-LacZ+ cells (b'). c Wing discs expressing GFP driven by C96-Gal4 and LacZ by wg-LacZ reporter. Scale bar, 20 μm. [file 12861_2020_217_MOESM4_ESM.jpg]

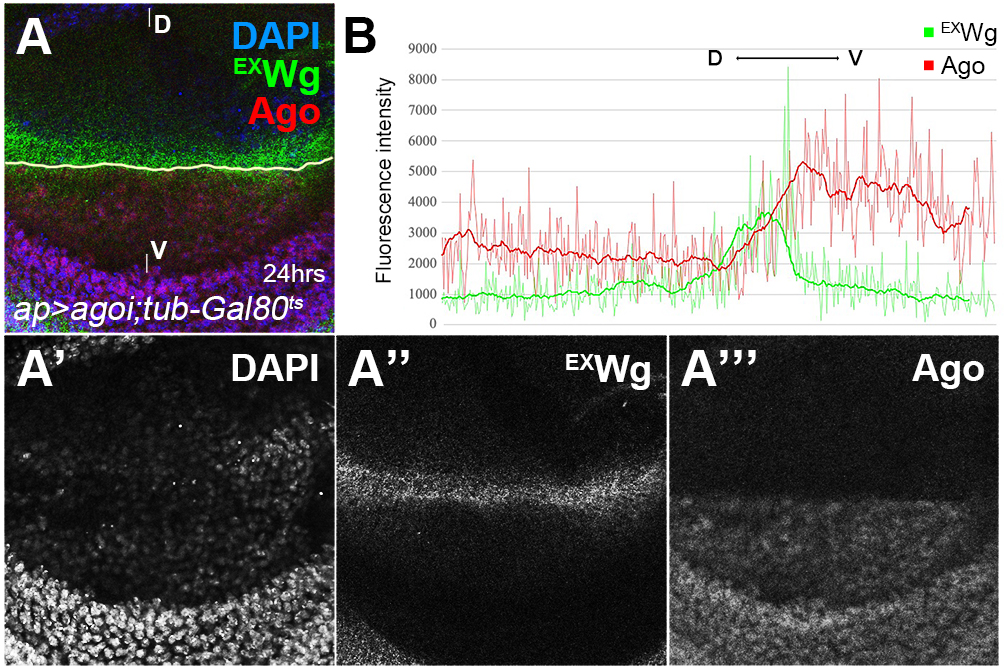

Supplement: Supplementary file 5 — Additional file 5: Figure S5. Knockdown of Ago reduces the steepness of Wg gradient. ago RNAi was induced for 24 h by ap-Gal4; tub-Gal80ts. a Wing discs were obtained immediately after Gal4 induction and stained for extracellular Wg. A representative image was presented (n=5). The composite image in (a) was individually shown in (a'-a'''). Yellow line distinguishes the DV midline based on anti-Ago (a'''). b The graph shows fluorescent intensity of extracellular Wg (green line) and Ago (red line) crossing DV midline marked in (a). [file 12861_2020_217_MOESM5_ESM.jpg]
